# Dissociation Between Neuronal and Astrocytic Calcium Activity in Response to Locomotion in Mice

**DOI:** 10.1093/function/zqad019

**Published:** 2023-04-29

**Authors:** Anna Fedotova, Alexey Brazhe, Maxim Doronin, Dmytro Toptunov, Evgeny Pryazhnikov, Leonard Khiroug, Alexei Verkhratsky, Alexey Semyanov

**Affiliations:** Faculty of Biology, Moscow State University, Moscow 119991, Russia; Department of Molecular Neurobiology, Shemyakin-Ovchinnikov Institute of Bioorganic Chemistry RAS, Moscow 117997, Russia; Faculty of Biology, Moscow State University, Moscow 119991, Russia; Department of Molecular Neurobiology, Shemyakin-Ovchinnikov Institute of Bioorganic Chemistry RAS, Moscow 117997, Russia; Department of Molecular Neurobiology, Shemyakin-Ovchinnikov Institute of Bioorganic Chemistry RAS, Moscow 117997, Russia; College of Medicine, Jiaxing University, Jiaxing, Zhejiang Province, 314001, China; Neurotar, Viikinkaari 4, FI-00790 Helsinki, Finland; Neurotar, Viikinkaari 4, FI-00790 Helsinki, Finland; Neurotar, Viikinkaari 4, FI-00790 Helsinki, Finland; Department of Forensic Analytical Toxicology, School of Forensic Medicine, China Medical University, Shenyang 110122, China; Faculty of Biology, Medicine and Health, The University of Manchester, Manchester M13 9PL, UK; Achucarro Centre for Neuroscience, IKERBASQUE, Basque Foundation for Science, 48011 Bilbao, Spain; Department of Stem Cell Biology, State Research Institute Centre for Innovative Medicine, LT-01102 Vilnius, Lithuania; Faculty of Biology, Moscow State University, Moscow 119991, Russia; Department of Molecular Neurobiology, Shemyakin-Ovchinnikov Institute of Bioorganic Chemistry RAS, Moscow 117997, Russia; College of Medicine, Jiaxing University, Jiaxing, Zhejiang Province, 314001, China; Department of Clinical Pharmacology, Sechenov First Moscow State Medical University, Moscow 119435, Russia

**Keywords:** astrocyte, neuron, Ca^2+^ signaling, locomotion, in vivo brain imaging; brain active milieu; paired-run ratio

## Abstract

Locomotion triggers a coordinated response of both neurons and astrocytes in the brain. Here we performed calcium (Ca^2+^) imaging of these two cell types in the somatosensory cortex in head-fixed mice moving on the airlifted platform. Ca^2+^ activity in astrocytes significantly increased during locomotion from a low quiescence level. Ca^2+^ signals first appeared in the distal processes and then propagated to astrocytic somata, where it became significantly larger and exhibited oscillatory behaviour. Thus, astrocytic soma operates as both integrator and amplifier of Ca^2+^ signal. In neurons, Ca^2+^ activity was pronounced in quiescent periods and further increased during locomotion. Neuronal Ca^2+^ concentration ([Ca^2+^]_i_) rose almost immediately following the onset of locomotion, whereas astrocytic Ca^2+^ signals lagged by several seconds. Such a long lag suggests that astrocytic [Ca^2+^]_i_ elevations are unlikely to be triggered by the activity of synapses among local neurons. Ca^2+^ responses to pairs of consecutive episodes of locomotion did not significantly differ in neurons, while were significantly diminished in response to the second locomotion in astrocytes. Such astrocytic refractoriness may arise from distinct mechanisms underlying Ca^2+^ signal generation. In neurons, the bulk of Ca^2+^ enters through the Ca^2+^ channels in the plasma membrane allowing for steady-level Ca^2+^ elevations in repetitive runs. Astrocytic Ca^2+^ responses originate from the intracellular stores, the depletion of which affects subsequent Ca^2+^ signals. Functionally, neuronal Ca^2+^ response reflects sensory input processed by neurons. Astrocytic Ca^2+^ dynamics is likely to provide metabolic and homeostatic support within the brain active milieu.

## Graphical Abstract

**Graphical Abstract:**
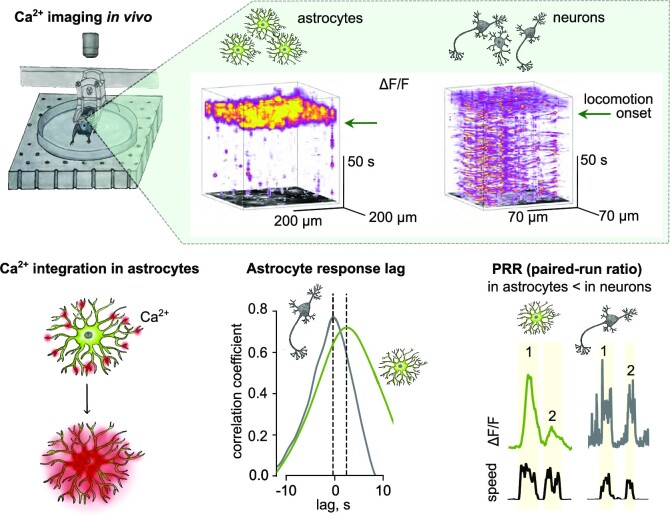

## Introduction

Brain function depends on closely coordinated interactions between different cell types, including neurons, glial cells, cells of the blood vessels, and noncellular elements such as extracellular space and extracellular matrix. This system of brain elements and their interactions is called the brain active milieu.^1,2^ The activity of electrically excitable neurons and their synaptic communication have been routinely recorded with electrode-based methods, demonstrating neuronal contribution to major brain functions: locomotion,^3^ sensory activity,^4^ vision,^5^ learning, and memory.^6^ Eclectically nonexcitable elements of the brain active milieu, including neuroglia, cannot be fully assessed with electrode-based techniques and require alternative approaches such as real-time optical imaging.

Optical methods allow monitoring of various types of cellular activity in the brain that include ionic signaling,^7–10^ metabolic processes,^11,12^ cellular morphology, and structure of extracellular space.^13–15^ Ca^2+^ imaging is the most popular: Multiple genetically encoded sensors were developed to monitor [Ca^2+^]_i_ in specific cell types.^16–18^

Two-photon microscopy provides cellular and subcellular resolution for imaging with genetically encoded sensors in vivo.^19–21^ However, combining this technique with animal behavior studies is difficult because the animal is kept in a head-fixed restrained condition throughout the experiment. Thus, experiments have been routinely performed on treadmills and air-floating balls that keep animals in a fixed position while allowing a certain type of locomotion.^22,23^ These methods can be combined with a virtual reality environment to design various behavioral paradigms.^24,25^ However, whether virtual reality faithfully represents the real world as an experimental animal perceives it remains an open question. In addition, a head-restricted animal cannot turn and always runs in a single direction on treadmills. The air-floating ball allows an animal to turn. Yet, the animal’s posture differs from that of an animal running on a flat surface: It resembles climbing an object, with the tail and the hindlimbs positioned below the head and forelimbs.

Using a flat airlifted platform rectifies the limitations of the above systems for 2-photon imaging in awake animals. This technique was used recently to monitor [Ca^2+^]_i_ dynamics in neurons and microglia in mice.^26,27^ However, the other elements of the brain active milieu have not been systematically studied in this experimental paradigm. Here, we compared the Ca^2+^ activity of astrocytes and neurons of mice navigating an airlifted platform.

## Materials and Methods

### Animals

All procedures were performed in accordance with the local guidelines for animal care (The Finnish Act on Animal Experimentation), FELASA ethical recommendations, NIH Guide for the Care and Use of Laboratory Animals, and the animal ethics and welfare committee of Jiaxing University. The C57BL/6 male (*N* = 6) mice were used in 2-photon imaging experiments. Mice were kept under standard housing conditions with a 12-h light/dark cycle (lights on at 9 am) with access to water and food ad libitum. Each animal underwent surgery at the age of 12–16 wk, followed by habituation to the experimental setup and imaging sessions. All experiments were carried out during the light period between 10 am and 5 pm.

### Adeno-associated Virus (AAV) Injection and Cranial Window Implantation

An analgesic (ketoprofen, 2.5 mg/kg) was administered to mice 30 min before the surgery and 24 h postsurgery. Mice were anaesthetized with a mixture of oxygen and isoflurane 1%–1.5% and placed inside the stereotaxic frame apparatus (RWD, Shenzhen, China). To maintain the animal’s body temperature at 37.0°C, a heating pad was used. An eye ointment was applied to the eyes to keep them moist during surgery. The head skin was removed, and a 3-mm craniotomy above the somatosensory cortex was performed using a dental drill (RWD, Shenzhen, China). Throughout the procedure, the skull was kept moist with applications of sterile saline. The stereotaxic injection of AAV5-gfaABC1D-cyto-GCaMP6f (Addgene viral prep #52925-AAV5), titer 10^11^ GC/mL, was performed to get an astrocyte-specific expression of genetically encoded Ca^2+^ indicator GCaMP6f in the somatosensory cortex (AP −2.3, ML +0.5, DV −0.6). To obtain expression in neurons, we used AAV2/9-CaMKII-GCaMP6f, titer 10^12^ GC/mL (VectorBuilder, Guangzhou, China). The total volume of 500 nL was injected via a glass pipette of 5 µL volume (Drummond, USA) using the automatic pump at the rate of 2 nL/s (Ultra Micro Pump III; World Precision Instruments, Sarasota, FL, USA). After each viral injection, the pipette tip was held for 10 min to ensure proper AAV diffusion in the region of interest. Next, 4-mm round cover glass (Warner Instruments, USA) was sealed to the skull with biocompatible adhesive (Vetbond, World Precision Instruments, Sarasota, FL, USA) and universal resin cement RelyX (3M, China). A lightweight stainless steel head plate (model 2, Neurotar, Helsinki, Finland) was mounted on the head with dental acrylic (Super-Bond C&B, Sun Medical, Japan) to restrain a mouse in the experimental setup under the objective of a 2-photon microscope (Femtonics, Budapest, Hungary). Dexamethasone (2 mg/kg) was administered to mice by subcutaneous injection to reduce the surgery-induced inflammation.

### Animal Habituation and Training

Before the imaging sessions, mice were handled and habituated to the experimental setup. Habituation started 3–4 wk after the surgery when the animals fully recovered. Awake mice were head-fixed under a microscope using a Mobile HomeCage device (Neurotar, Helsinki, Finland). Mice heads were secured in the clamping mechanism, and the device was connected to a pressurized air source. Once the carbon fiber cage began to float, animals could move it with their paws. Animals were habituated to head fixation in four to six 2-h training sessions repeated once per day.

### Ca^2+^ Imaging

After habituation and training, mice were used for Ca^2+^ imaging. Multiphoton fluorescence microscope (Femtonics, Budapest, Hungary) with a 16× NA 0.8 water-immersion objective (CFI75 LWD 16X W, Nikon, Tokyo, Japan) or with a 20× NA 0.8 water-immersion objective (XLUMPLFLN20XW, Olympus, Tokyo, Japan) was used for Ca^2+^ imaging in vivo. Ti:Sapphire laser (Chameleon Ultra, Coherent, USA) with an excitation wavelength of 920 nm was used to excite the fluorescence of GCaMP6f. Fluorescence signal from a brain area of 600 × 600 or 175 × 175 µm^2^ (512 × 512 pixels) was recorded in resonant scanning mode at 30 frames/s. The signal was filtered with a 520/60 nm bandpass filter (Semrock, Rochester, NY, USA) and then detected with a GaAsP photomultiplier (H11706P-40, Hamamatsu, Japan). Simultaneously, autofluorescence was filtered with a 650/100 nm bandpass filter and detected with a second identical photomultiplier. Several imaging sessions of 10 min were carried out for each mouse. In parallel with Ca^2+^ imaging, the animal movements on the platform were monitored with locomotion-tracking software (Neurotar, Helsinki, Finland). All sessions were video recorded in infrared light. The experimental session did not exceed 2 h per mouse.

### Processing Ca^2+^ Imaging Data

A few procedures and basic estimated parameters were the same for astrocytic and neuronal Ca^2+^ data. At the same time, some extracted characteristics differed to match the disparities in the responses of the 2 cell types. Preprocessing steps were identical for both astrocytic and neuronal data. Most of the image processing was done using routines from Image-funcut, uCats, and other software developed in the group using the common scientific Python library stack (SciPy, Scikit-learn, Scikit-image).^28,29^

### Preprocessing: Temporal Binning, Motion Correction, and Denoising

The raw imaging data recorded at 30 Hz were binned along a time dimension by summing intensity values in nonoverlapping batches of 5 frames before further processing to increase the signal-to-noise ratio. Next, rigid-body motion correction was applied using an autofluorescence signal recorded in the red channel as a morphology marker. For a more stable shift estimation, the autofluorescence signal was adaptively denoised by approximating each frame by the first 50 spatial principal components, smoothed by a Gaussian filter with σ = 1 pixel. The spatial displacements estimated for the autofluorescence channel have then been applied to the green channel containing GCaMP6f fluorescence.

After shift correction, the data were further denoised using the algorithm developed in the lab. In brief, the data were Anscombe transformed to stabilize variance, and then the recording was divided into overlapping spatial windows (patches), where the data are approximated by truncated SVD with a smoothing filter applied to the spatial components, which is then followed by inverse SVD and Anscombe transforms, averaging the approximations in the overlapping parts of the patches.

### Calculation of Δ*F/F* and Active Area

Slowly varying fluorescent baseline level *F*_0_ was determined as a lower envelope running through local minima of the fluorescence signal in each pixel, smoothed by a Gaussian filter with σ = 15 s. The resulting baselines were negatively biased, and to correct this bias, the baseline signals in each pixel were then shifted by a constant parameter minimizing the absolute value of the mode of the residuals after baseline subtraction, assuming that most frequent fluctuations are small and are due to noise, while true Ca^2+^ events are sparse. Further analysis is done on relative fluorescence changes Δ*F/F = (F−F*_0_*)/F*_0_*×* 100%. An arbitrarily chosen threshold of 15% increase over the baseline (Δ*F/F* ≥ 15%) was selected as it provided the most visually informative results. Clusters of suprathreshold pixels larger than 16 pixels within a frame were marked as an “active segment” if they produced overlapping structures in at least 3 consecutive frames. The fractional area of such active segments in each frame normalized to the total stained area within the field of view was named “active area” and used to characterize the collective Ca^2+^ activity.

### Analysis Specific to Astrocytes

Individual astrocytic domains were visually identified and manually traced. Inside the domain territory, the soma was likewise outlined. The rest of the domain was called processes. Δ*F/F* timecourses were plotted for both processes and soma. Then, the timecourses were normalized to their peak values. The latency between soma and processes was estimated as the difference between the times when Δ*F/F* reached 15% of the maximal value. The prominence of Δ*F/F* oscillations was estimated by averaging wavelet power in the interval when the signal was above 15% of the peak value in the frequency range 0.1–0.3 Hz (continuous wavelet transform, Morlet wavelet).

### Analysis Specific to Neurons

Because neuronal Ca^2+^ data displayed a high spatially localized spontaneous activity, we employed a slightly modified constrained non-negative matrix decomposition (CNMF) approach to visualize this activity during locomotion.^30^ CNMF is initially optimized for extracting Ca^2+^ dynamics from cell bodies, but lifting temporal dynamics and spatial localization constraints can allow for the segmentation of sparse components. The algorithm was applied in overlapping 100 × 100 patches with 12 components per patch to solve for. A second run of the algorithm on the entire frame with the patch-based components as the initial condition was then applied to refine the segmentation. The resulting spatial components could still contain irregular low-amplitude shapes, which were clipped by using Li’s auto threshold algorithm on nonzero pixels in each component.^31^ The resulting solution produced many spatial segments with similar dynamics, further merged by clustering their corresponding dynamics (agglomerative clustering with Ward linkage) to produce 12 final spatial regions of interest (ROIs).

### Statistical Analysis

Data are presented as median [25th–75th percentiles]. The sample number (*n*) indicates the number of recordings, periods of locomotion or quiescence, or cells and is specified for each experiment. Astrocytic Ca^2+^ activity was recorded in 2 mice and neuronal in 4 mice. Statistical analysis was conducted using Python. For comparison of astrocytic and neuronal Ca^2+^ activity (Δ*F/F* and active area) during the periods of quiescence and locomotion, the nonparametric Mann–Whitney *U*-test was used. For other comparisons, linear mixed-effects models were applied.^32^ The experimenters were not blinded to the experimental conditions, and no randomization was performed. All statistical details of the experiments are provided in the main text and figure legends. *P* < .05 was considered statistically significant.

## Results

### Ca^2+^ Activity in Cortical Astrocytes Is Linked to Locomotion but Not Location

**Figure 1.**
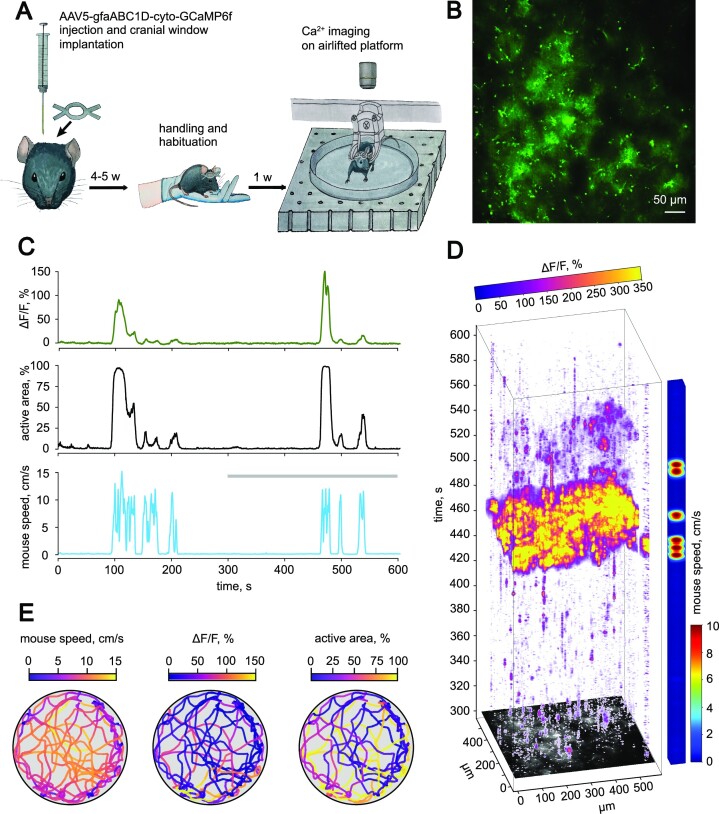
Properties of Ca^2+^ activity in cortical astrocytes during locomotion. (A) Experimental design: AAV5-gfaABC1D-cyto-GCaMP6f was injected into the somatosensory cortex of a C57Bl/6 mouse, followed by chronic cranial window implantation. After 3–4 wk of GCaMP6f expression, mice were handled and habituated to the experimental setup. Ten-minute Ca^2+^ imaging sessions combined with animal tracking were performed after the training procedure. (B) The fluorescence signal from cortical astrocytes expressing GCaMP6f in an awake mouse. (C) The timecourses of Δ*F/F* (top), the active area (middle), and the mouse speed (bottom). The gray line indicates the part of the recording used for the reconstruction shown in panel (D). (D) The 3-dimensional reconstruction (*x–y* time) of Δ*F/F* values showing the distribution of astrocytic Ca^2+^ activity over the imaged region in time. The colored bar on the right-hand side encodes the speed of the animal. (E) The animal track recorded for 600 s color-coded with the mouse speed (left), Δ*F/F* (middle), and the active area (right).

The somatosensory cortex area of mice containing projections of the left forelimb was injected with an AAV for expression of genetically encoded Ca^2+^ sensor GCaMP6f under either astrocytic (AAV5-gfaABC1D-cyto-GCaMP6f) or neuronal (AAV2/9-CamKII-GCaMP6f) promotors (Figure 1A). The cranial glass window was implanted above the site of injection. A lightweight stainless steel head plate was glued above the window. After recovery from the surgery, the animals were left for 3–4 wk in individual cages for GCaMP6f expression. After handling the animals and their habituation to the airlifted platform, Ca^2+^ imaging experiments were performed (Video S1).

First, the imaging was done on astrocytes expressing GCaMP6f in cortical layer 1 (Figure 1B; Video S2). The animal locomotion episodes alternated with quiescence periods (Figure 1C and D). During quiescent periods, Ca^2+^ activity appeared as small, scattered events.^10^ Jointly, these events covered only a tiny frame area (mean active area per period of quiescence: 1.0 [0.8–1.7]% of frame; *n* = 21) and had low Δ*F/F* (mean Δ*F/F* per period of quiescence: 1.4 [1.0–1.6]%; *n* = 21). Consistent with previous reports, the locomotion was accompanied by a rise in astrocytic [Ca^2+^]_i_.^20,33,34^ We observed an increase in both the active area (mean active area per episode of locomotion: 20.2 [10.7–36.1]% of frame; *n* = 29; *P* < .001, mixed-effects model) and Δ*F/F* (mean Δ*F/F* per episode of locomotion: 9.9 [6.3–19.2]%; *n* = 29; *P* < .001, mixed-effects model). Sometimes, the animal ran several times with short intervals of quiescence. Astrocytic [Ca^2+^]_i_ increased during each running episode, but this increase became smaller in subsequent episodes.

Astrocytic Ca^2+^ imaging was combined with animal tracking (Figure 1E). The mouse ran for several seconds in each episode and moved around the entire territory of the platform during the experiment. However, the animal preferred to rest (quiescence period) at the platform’s periphery. Ca^2+^ activity followed the locomotion pattern without an obvious link to the animal’s location.

### Integrative Function of Astrocytes

**Figure 2.**
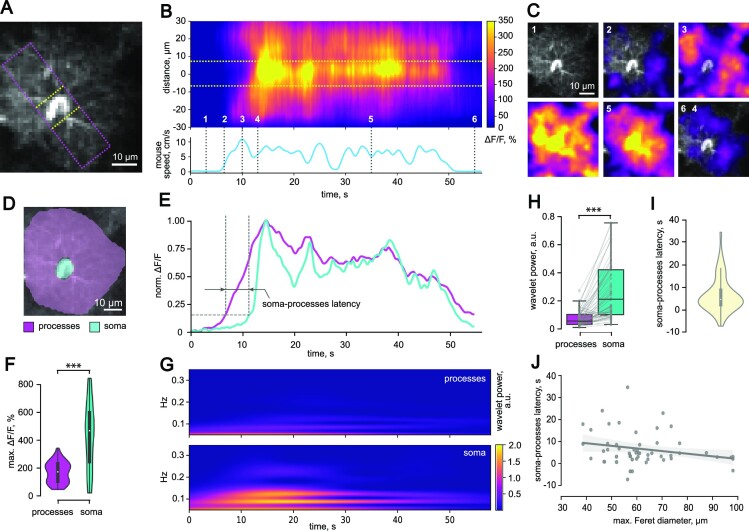
Locomotion-induced Ca^2+^ activity in a single astrocyte. (A) An astrocyte image with the position of a wide line-scan (pink rectangle). Yellow dotted lines indicate the soma. (B) (Top) The fluorescence line-scan (x–t) image showing Ca^2+^ response to locomotion in the astrocytic processes and the soma. The distance was calculated from the center of the soma (0 µm). The yellow dotted lines mark the width of the soma. (Bottom) The timecourse of mouse speed during the locomotion episode corresponding to the above line-scan. (C) The spatial distribution of Ca^2+^ activity (Δ*F/F*) for the time points indicated in (B) by the dotted lines. (D) An astrocyte segmented to the soma and the processes. (E) The timecourses of Ca^2+^ activity (Δ*F/F*) normalized to the maximal Δ*F/F* in the astrocytic soma and processes during the episode of locomotion shown at (B). Soma-processes latency was calculated between the points corresponding to 15% of the maximal Δ*F/F* (dotted lines and arrows). (F) Summary data of maximal Δ*F/F* in the processes and the somata (***P < .001, mixed-effects model, *n* = 53 cells). (G) The wavelet spectrograms of the fluorescent signals shown in (E). (H) Average wavelet power (a.u.) taken over interval when ΔF/F in the astrocytic processes (top) and soma (bottom) was above 15% of the peak and within 0.1–0.3 Hz frequency range (***P < .001, mixed-effects model, *n* = 53 cells). (I) The distribution of soma-processes latencies of Ca^2+^ responses. (J) The relationship between soma-processes latency of Ca^2+^ responses and astrocyte size expressed as maximum Feret diameter (Regression analysis: *R*^2^ = 0.055, *P* = .09, linear regression, *n* = 53 cells).

Spontaneous Ca^2+^ events are primarily localized to distal astrocytic processes.^35,36^ We observed that during locomotion, [Ca^2+^]_i_ increased in the entire astrocytic domain, including soma. This observation prompts a question of whether Ca^2+^ activity initiated in soma subsequently diverges to astrocytic processes or [Ca^2+^]_i_ rises in distal processes propagate toward the soma. We performed a wide line-scan across the entire astrocytic domain and then analyzed [Ca^2+^]_i_ changes in soma and processes (Figure 2A). During the mouse running episode [Ca^2+^]_i_ elevations started in distal parts of the astrocytic domain and then propagated to the soma (Figure 2B). This phenomenon was also observed in the consecutive frames showing Δ*F/F* over cell morphology image (Figure 2C). Notably, Ca^2+^ response also first faded in the soma at the end of the running episode.

Next, we compared the timecourses of Δ*F/F* sampled from the soma and the processes (Figure 2D and E). First, maximal Δ*F/F* was significantly larger in the soma (processes: 170.3 [102.8–229.7]%, *n* = 53; soma: 465.0 [245.1–595.3]%; *n* = 53; *P* < .001, mixed-effects model; Figure 2F). Second, we observed that periodic [Ca^2+^]_i_ oscillations, which were more pronounced in soma than in astrocytic processes (wavelet power; processes: 0.06 [0.03–0.10] a.u., *n* = 53; soma: 0.21 [0.10–0.42] a.u.; *n* = 53; *P* < .001, mixed-effects model; Figure 2G and H). Finally, we measured the latency between Δ*F/F* increases in the soma and the processes (4.7 [2.3–8.9] s, *n* = 53; Figure 2I). Because the latency varied in a wide range, we tested if this variability can be explained by the variability in astrocyte domain size. However, we did not find a significant correlation between the latency and the astrocyte maximum Feret diameter (*R*^2^ = 0.055, *P* = .09, linear regression, *n* = 53; Figure 2J).

These results suggest that Ca^2+^ signals start in astrocytic processes and converge into soma during animal locomotion. Astrocytic soma integrates the Ca^2+^ signal, amplifies it, and generates Ca^2+^ oscillations.

### Neuronal Ca^2+^ Response to Locomotion

**Figure 3.**
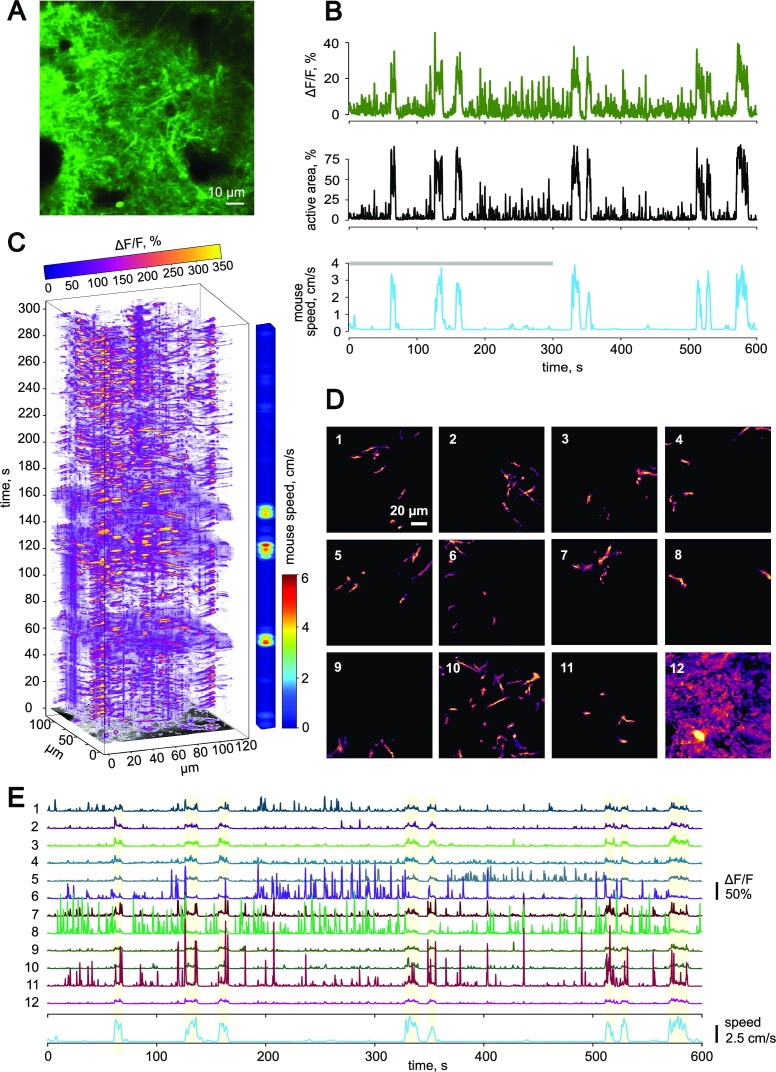
Properties of Ca^2+^ activity in cortical neurons during locomotion. (A) The fluorescence signal from cortical neurons expressing GCaMP6f in an awake mouse. (B) The timecourses of Δ*F/F* (top), the active area (middle), and the mouse speed (bottom). The gray line indicates the part of the recording used for the reconstruction shown in (C). (C) The 3-dimensional reconstruction (*x–y* time) of Δ*F/F* values showing the distribution of neuronal Ca^2+^ activity over the imaged region in time. The colored bar on the right side encodes the speed of the animal. (D) ROIs or units of synchronous neuronal Ca^2+^ activity segmented with CNMF-based approach within the recording presented at (B and C). (E) The timecourses of Ca^2+^ activity (Δ*F/F*) in individual ROIs (activity units) shown and numbered at (D). The blue timecourse at the bottom is animal speed. Light yellow-shaded regions represent episodes of locomotion.

Neuronal Ca^2+^ activity was recorded in cortical layer 1 (Figure 3A). In this layer, we rarely saw somata of the neurons but observed multiple processes. Some of these processes can be visually identified as dendrites with spines, and others cannot be classified into any specific type (dendrites or axons). Many neuronal processes exhibited high Ca^2+^ activity during quiescent periods (Figure 3B and C; Video S3). Thus, unlike astrocytes, the neurons were active during locomotion and quiescent periods.

During quiescent periods, the mean active area was 6.0 [4.4–9.4]% of the frame (*n* = 37), and the mean Δ*F/F* per period of quiescence was 3.1 [2.7–4.2]% (*n* = 37). Locomotion was accompanied by a rise in neuronal [Ca^2+^]_i_ (mean active area per episode of locomotion: 53.4 [43.8–62.2]%, *n* = 29; *P* < .001, mixed-effects model; mean Δ*F/F* per episode of locomotion: 19.5 [16.5–26.8]%, *n* = 29; *P* < .001, mixed-effects model).

To identify how different neuronal compartments contribute to Ca^2+^ dynamics during quiescent periods and locomotion, we applied a constrained non-negative matrix factorization (CNMF) approach implemented in CaImAn, open-source software for Ca^2+^ imaging analysis.^37^ With CNMF followed by clustering, we identified neuronal structures activated synchronously within individual recordings (Figure 3D). These structures represented separate neuronal processes. We cannot confidently conclude whether these processes belong to the same neuron; thus, we considered them activity units. We analyzed each activity unit by plotting the corresponding [Ca^2+^]_i_ timecourses (Figure 3E). Such analysis revealed 2 types of units, which had either high activity or low activity during quiescent periods. In the low activity, units [Ca^2+^]_i_ increased during locomotion. In the high activity, units [Ca^2+^]_i_ did not increase and in some cases decreased. Also, we noticed that the [Ca^2+^]_i_ increased during locomotion in the regions where we could not unequivocally identify neuronal structures. These regions may correspond to the out-of-focus or dim structures.

### Dissociation Between Neuronal and Astrocytic Ca^2+^ Response to Locomotion

**Figure 4.**
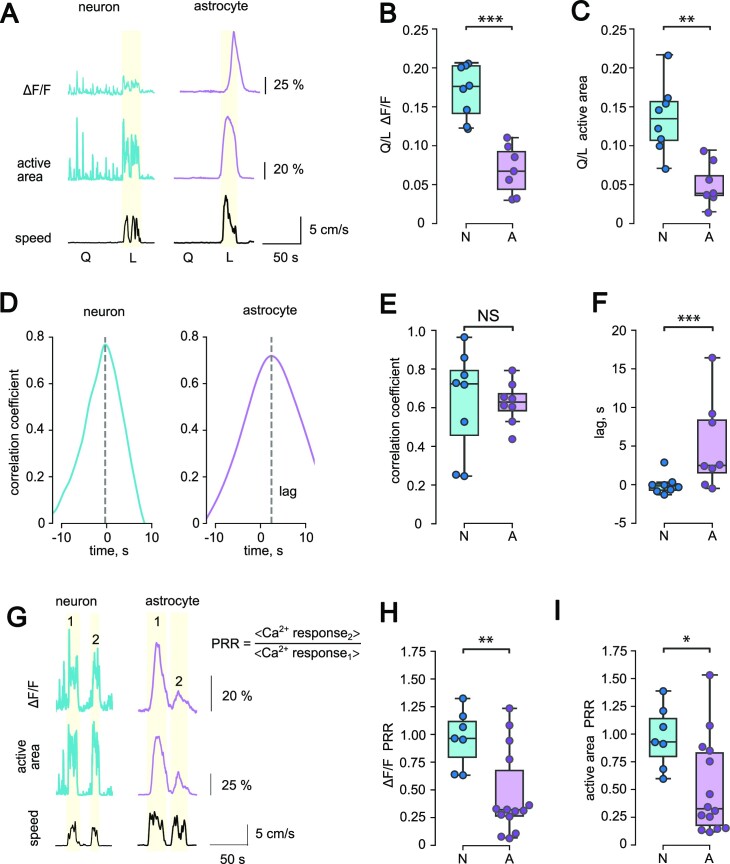
Comparison of locomotion-induced Ca^2+^ responses in neurons and astrocytes. (A) The timecourses demonstrating changes in Δ*F/F* (top) and active area (middle) of neuronal (left) and astrocytic (right) Ca^2+^ activity during the periods of quiescence (Q) and locomotion (L). (Bottom) The timecourse of animal speed. (B and C) Q/L ratio, calculated for Ca^2+^ activity in cortical neurons (“N,” *n* = 8 recordings) and astrocytes (“A,” *n* = 7 recordings; ***P < .001 and **P = .001 for Δ*F/F* (B) and active area (C), respectively, Mann–Whitney *U*-test). (D) Cross-correlation graphs between ΔF/F and animal speed for neurons (left) and astrocytes (right) during an episode of locomotion. (E) Peak correlation coefficients for neurons (“N,” *n* = 8 recordings) and astrocytes (“A,” *n* = 7 recordings; *P* = .87, mixed-effects model) for the analysis presented at (D). (F) Lag of cross-correlation peak in neurons (“N,” *n* = 8 recordings) and astrocytes (“A,” *n* = 7 recordings; ***P < .001, mixed-effects model). (G) The timecourses demonstrating changes in ΔF/F (*top*) and active area (middle) of neuronal (left) and astrocytic (right) Ca^2+^ activity during pairs of locomotion episodes. (Bottom) The timecourse of animal speed. The formula is for PRR calculation where < Ca^2+^ response_1_ > is the mean Ca^2+^ response to the first locomotion in 1 recording; <Ca^2+^ response_2_ > is the same for the second locomotion. (H and I) PPRs for Δ*F/F* (**P = .002, mixed-effects model) and active area (*P = .014, mixed-effects model) in neurons (“N,” *n* = 7 paired runs) and astrocytes (“A,” *n* = 14 paired runs).

Next, we compared the parameters of Ca^2+^ responses to locomotion between astrocytes and neurons. First, we measured mean Δ*F/F* during the quiescent period (Q) and locomotion (L). The Q/L ratio of Δ*F/F* was significantly higher in neurons than in astrocytes (in neurons: 0.18 [0.14–0.20], *n* = 8; in astrocytes: 0.067 [0.044–0.092], *n* = 7; *P* < .001, Mann–Whitney *U*-test; Figure 4A and B). The Q/L ratio calculated for the active area was also significantly higher in neurons (in neurons: 0.13 [0.11–0.16], *n* = 8; in astrocytes: 0.039 [0.0036–0.061], *n* = 7; *P* = .001, Mann–Whitney *U*-test; Figure 4C). These results reflect higher Ca^2+^ activity in neurons during quiescent periods as compared to astrocytes.

Second, we asked when Ca^2+^ response in neurons and astrocytes develops relative to locomotion. We performed cross-correlations of locomotion speed and [Ca^2+^]_i_ elevations (Δ*F/F*) in neurons and astrocytes. The cross-correlations yielded reasonably high peak coefficients in both cell types (in neurons: 0.72 [0.46–0.79], *n* = 8; in astrocytes: 0.63 [0.59–0.67], *n* = 8; *P* = .87, mixed-effects model; Figure 4D and E). In neurons, the time of the peak was around 0, suggesting that locomotion and [Ca^2+^]_i_ elevation coincided (−0.16 [−0.67–0.08] s, *n* = 8). In astrocytes, the peak had a lag of several seconds (2.5 [1.6–8.3], *n* = 8; *P* < .001, mixed-effects model; Figure 4F). This result indicates that Ca^2+^ response in astrocytes develops several seconds after the beginning of the locomotion episode and neuronal Ca^2+^ response.

Finally, we compared neuronal and astrocytic responses to 2 consecutive episodes of locomotion. We selected pairs of closely timed running episodes with less than 30 s intervals. Then, we calculated the paired-run ratio (PRR) as the ratio between the peaks of second and first Ca^2+^ responses (Δ*F/F* or active area) corresponding to 2 locomotion episodes (Figure 4G). The PRR of Δ*F/F* in neurons was close to 1 (0.96 [0.80–1.11], *n* = 7; Figure 4H) suggesting that neurons reliably respond to each locomotion episode. Conversely, PRR of Δ*F/F* in astrocytes was significantly lower than in neurons (0.32 [0.267–0.67), *n* = 14; *P* = .002, mixed-effects model) suggesting a decline in astrocytic response. Similar results were obtained for PRR of active area (in neurons 0.93 [0.80–1.14], *n* = 7; in astrocytes 0.33 [0.18–0.83], *n* = 14; *P* = .014, mixed-effects model; Figure 4I). These findings demonstrate that neuronal Ca^2+^ activity reliably increases with every locomotion episode. In contrast, astrocytic Ca^2+^ signal has refractoriness that prevents Ca^2+^ response from developing during closely timed locomotion episodes.

## Discussion

### The Distinct Timecourses of Neuronal and Astrocytic Ca^2+^ Response to Locomotion

Cortical neurons receive sensory input from thalamic nuclei. During locomotion, neuronal firing triggers Ca^2+^ entry through voltage-gated Ca^2+^ channels. We observed that overall neuronal Ca^2+^ activity in the somatosensory cortex increased during animal locomotion. However, neurons remained active in quiescent periods between episodes of locomotion. In some neuronal units, activity during quiescence was higher than that during locomotion.

Local [Ca^2+^]_i_ elevations in astrocytic leaflets can be mediated by Ca^2+^ entry through the plasma membrane (through ionotropic receptors or reversed Na^+^/Ca^2+^ exchanger), whereas large Ca^2+^ transients in soma and branches are mediated by Ca^2+^ release from endogenous stores—endoplasmic reticulum and mitochondria.^38–40^ During animal quiescence, astrocytes generated small, scattered Ca^2+^ events. These events can be either spontaneous or triggered by local synaptic activity. During locomotion, astrocytic Ca^2+^ activity substantially increased. Relative to Ca^2+^ activity during the quiescent period, this increase was significantly larger in astrocytes than in neurons. Moreover, astrocytic [Ca^2+^]_i_ elevation was delayed for several seconds after the beginning of the locomotion episode and neuronal [Ca^2+^]_i_ elevation. These findings suggest that the activity of the local neuronal network may not trigger the astrocytic Ca^2+^ signal. Most likely, astrocytic response originates from modulatory subcortical projections, such as noradrenergic input from the *locus coeruleus* targeting adrenergic receptors abundantly expressed in mature astrocytes.^41–43^ Delayed astrocytic Ca^2+^ response also raises a question of its physiological relevance. This delay suggests that astrocytes do not respond to sensory signals in real time, hence are involved in the real-time processing of sensory information. On the other hand, astrocytic Ca^2+^ activity may be involved in modulation of synaptic plasticity,^44^ regulation of vascular tone,^45^ and metabolic activation of astrocytes^46^ that occur with a delay. For example, synaptic plasticity and memory consolidation may happen after sensory information is processed, filtered, and compared to previous memories in the brain. The same applies to vasodilatation and metabolic activation of astrocytes, which can represent a systemic response to the increased energy demand of the brain following robust neuronal activity during locomotion.^47^

### A Refractory Period of Ca^2+^ Activity in Astrocytes

Another example of dissociation between Ca^2+^ activity in the local neuronal network and astrocytes is highlighted by distinct changes in the PRR. When an animal ran twice with a short interval (<30 s), the neuronal network reliably responded to each locomotion episode. Astrocytes responded with a large Ca^2+^ transient only to the first run. The response to the second run was significantly diminished. Most likely, it is related to the mechanisms of Ca^2+^ signal generation in these 2 cell types. In neurons, the bulk of Ca^2+^ enters through ligand- or voltage-gated channels of the plasma membrane.^48,49^ Because the transmembrane Ca^2+^ gradient is quickly restored, neurons are ready to respond to the second run. Global astrocytic Ca^2+^ transients depend on Ca^2+^ release through inositol 1,4,5-trisphosphate receptors (InsP_3_R) from the endoplasmic reticulum, which, upon strong stimulation, gets depleted, while InsP_3_R become inactivated.^39,40,50^ It takes substantially more time to restore the ability to generate Ca^2+^ signals for astrocytes than for neurons. What is the physiological relevance of such a refractory period? Astrocytic Ca^2+^ stimulates oxidative phosphorylation.^51^ However, due to the sparse distribution of cytochromes in the astrocytic electron transport chain, there is also a high probability of reactive oxygen species (ROS) generation in response to [Ca^2+^]_i_ elevation in astrocytes.^52,53^ In moderate quantities, ROS play a signaling role and promote synaptic plasticity in the brain.^54^ In high quantities, they cause oxidative stress and cell damage. Therefore, the astrocytic refractory period may preserve ROS’s beneficial effects while preventing cell damage.

### Integrative Function of Astrocytes

At the subcellular level, [Ca^2+^]_i_ did not increase simultaneously in the entire astrocytic morphological domain during locomotion. [Ca^2+^]_i_ elevation started in the distal processes and then propagated into the soma. This finding is consistent with a higher probability for Ca^2+^ activity generation in distal astrocytic processes.^35,55^ This phenomenon may be explained by the specific localization of synapses near distal astrocytic processes. However, a recent report demonstrated a relatively even distribution of synapses near distal processes and astrocytic soma.^56^ An alternative explanation is that distal astrocytic processes have a higher surface-to-volume ratio (SVR).^36^ High SVR increases the amplitudes of [Ca^2+^]_i_ elevations induced by Ca^2+^ entry through the plasma membrane and consequently increases the probability of Ca^2+^-dependent Ca^2+^ release from endogenous stores.

Our observations also suggest that astrocytic soma operates as an integrator of Ca^2+^ activity starting in the processes. Ca^2+^ signal integration in astrocytic soma is reminiscent of the integration of membrane voltage changes from dendritic inputs by soma in neurons.^57^ However, in the case of neurons, somatic integration is translated into a pattern of action potentials. What is the functional relevance of Ca^2+^ activity integration in astrocytes? One possibility is an amplification of the Ca^2+^ signals. The Ca^2+^ response in soma was several fold higher than that in the processes. Different levels of [Ca^2+^]_i_ regulate distinct molecular pathways.^58^ Thus, Ca^2+^ amplification may target specific cellular functions. For example, somatic [Ca^2+^]_i_ elevation can boost ATP production by promoting the activity of the tricarboxylic acid cycle enzymes, the proteins of the electron transport chain, and the ATP synthase.^59,60^ Such enhanced metabolic response of astrocyte can be the physiological outcome of Ca^2+^ integration, homologues to, and yet distinct from, synaptic integration and action potential generation in neurons.

### Ca^2+^ Oscillations in Astrocytic Soma

In addition, Ca^2+^ integration in astrocytic soma triggers [Ca^2+^]_i_ oscillations. [Ca^2+^]_i_ oscillations in astrocytes were routinely recorded in primary cultures and in slices in response to pharmacological stimulation.^61–64^ Mathematical models of astrocytic [Ca^2+^]_i_ dynamics also predict oscillatory activity due to positive and negative feedbacks.^65,66^ Although astrocytes, in principle, should possess mechanisms for [Ca^2+^]_i_ oscillations, oscillatory behavior was not reported in vivo. A possible reason is that Ca^2+^ activity in astrocytes in vivo is defined by inputs from many elements of the brain active milieu (local neuronal network, neuromodulatory projections, blood vessels, etc.), which drive the sequence of astrocytic Ca^2+^ events. However, astrocyte oscillatory mechanisms can re-emerge during somatic [Ca^2+^]_i_ elevations. These oscillations originate specifically in the soma and fade away when propagating toward the astrocytic periphery. [Ca^2+^]_i_ oscillations require [Ca^2+^]_i_ to exceed a certain threshold. Large [Ca^2+^]_i_ elevations and well-expressed Ca^2+^ stores in the soma provide optimal conditions for [Ca^2+^]_i_ oscillations.^65^ Possibly, the information is encoded in the frequency of [Ca^2+^]_i_ oscillations that depends on the strength of the external signal and intrinsic properties of an individual astrocyte. The oscillatory Ca^2+^ signal is decoded by the enzymes containing Ca^2+^ binding motifs with a distinct affinity that can regulate their phosphorylation and activate specific cellular programs.^67^

## Conclusion

We demonstrate that Ca^2+^ dynamics in astrocytes does not follow neuronal Ca^2+^ activity that accompanies locomotion, indicating that astrocytic [Ca^2+^]_i_ is controlled by pathways distinct from sensory inputs that activate neuronal networks. While neuronal [Ca^2+^]_i_ faithfully follows episodes of animal locomotion, astrocytic Ca^2+^ signals develop on a different timescale, compatible with distinct roles astrocytes play in sustaining nervous tissue and supporting neuronal information processing and storage.

## Supplementary Material

zqad019_Supplemental_Movies

## Data Availability

The data underlying this article will be shared on reasonable request to the corresponding author.
